# 5-Hydroxymethylcytosine preferentially targets genes upregulated in isocitrate dehydrogenase 1 mutant high-grade glioma

**DOI:** 10.1007/s00401-018-1821-3

**Published:** 2018-02-10

**Authors:** Wioletta K. Glowacka, Harshika Jain, Makiko Okura, Abulizi Maimaitiming, Yasin Mamatjan, Romina Nejad, Hamza Farooq, Michael D. Taylor, Kenneth Aldape, Paul Kongkham

**Affiliations:** 1 MacFeeters-Hamilton Centre for Neuro-Oncology Research, University Health Network, Toronto, Canada; 2 Division of Neurosurgery, Toronto Western Hospital, University Health Network, 339 Bathurst St, WW4-450, Toronto, ON M5T-2S8 Canada; 3 Arthur and Sonia Labatt Brain Tumour Research Centre, The Hospital for Sick Children, Toronto, Canada; 4 Division of Neurosurgery, The Hospital for Sick Children, Toronto, Canada; 5 Department of Laboratory Medicine and Pathobiology, University Health Network, Toronto, Canada; 6 Division of Respiratory Medicine, Faculty of Medicine, Juntendo University, Tokyo, Japan

**Keywords:** 5-Hydroxymethylcytosine (5hmC), Glioma, EPIC BeadChip, G-CIMP, Enhancer, *IDH1* mutation

## Abstract

Gliomas demonstrate epigenetic dysregulation exemplified by the Glioma CpG Island Methylator Phenotype (G-CIMP) seen in *IDH1* mutant tumors. 5-Hydroxymethylcytosine (5hmC) is implicated in glioma pathogenesis; however, its role in *IDH1* mutant gliomas is incompletely understood. To characterize 5hmC in *IDH1* mutant gliomas further, we examine 5hmC in a cohort of *IDH1* mutant and wild-type high-grade gliomas (HGG) using a quantitative locus-specific approach. Regions demonstrating high 5hmC abundance and differentially hydroxymethylated regions (DHMR) enrich for enhancers implicated in glioma pathogenesis. Among these regions, *IDH1* mutant tumors possess greater 5hmC compared to wild type. 5hmC contributes to overall methylation status of G-CIMP genes. 5hmC targeting gene body regions correlates significantly with increased gene expression. In particular, a strong correlation between increased 5hmC and increased gene expression is identified for genes highly expressed in the *IDH1* mutant cohort. Overall, locus-specific gain of 5hmC targeting regulatory regions and associated with overexpressed genes suggests a significant role for 5hmC in *IDH1* mutant HGG.

**Electronic supplementary material:**

The online version of this article (10.1007/s00401-018-1821-3) contains supplementary material, which is available to authorized users.

## Introduction

Gliomas are the most common primary human brain tumor, with high-grade gliomas (HGG) of World Health Organization (WHO) grades III and IV being the most aggressive. Grade IV tumors (Glioblastoma, GBM) carry the worst prognosis with a median overall survival (OS) of approximately 15 months [61]. GBM may arise de novo or secondarily from lower grade gliomas (LGG). Recent molecular analyses through The Cancer Genome Atlas (TCGA) have identified four molecular GBM subtypes with unique gene expression, DNA copy number, and mutation profiles [70]. Among recurrent events is mutation of isocitrate dehydrogenase 1 (*IDH1*). *IDH1* mutation occurs in 12% of GBMs and with greater frequency among LGGs and secondary GBMs [54, 76]. In addition, *IDH1* mutation occurs predominantly in the proneural GBM subtype and is associated with the Glioma CpG Island Methylator Phenotype (G-CIMP) [10, 52].

*IDH1* mutation in glioma results in a gain of function, producing the oncometabolite 2-hydroxyglutarate (2-HG) [75]. 2-HG inhibits α-ketoglutarate-dependent enzymes including the Ten-Eleven Translocation (TET) family of DNA dioxygenases [75]. TET proteins play a critical role in active cytosine demethylation, oxidizing 5-methylcytosine (5mC) to 5-hydroxymethylcytosine (5hmC). Further oxidation steps generate 5-formylcytosine (5fC) and 5-carboxylcytosine (5caC), which are converted to cytosine through thymine DNA glycosylase base excision repair mechanisms [8, 66]. *IDH1* mutation-mediated inhibition of TET proteins is one mechanism altering the DNA methylation landscape in G-CIMP gliomas [68]. Traditional approaches to quantify methylation using bisulfite (BS) conversion strategies, including those used to define G-CIMP, do not distinguish 5mC from 5hmC [7, 52]. 5hmC is a stable epigenetic mark with evidence suggesting a role for 5hmC in regulating gene expression, beyond being simply a passive intermediate in the process of active demethylation [8, 41, 51, 59].

Loss of 5hmC is observed in multiple human malignancies [6, 19, 28, 35]. While 5hmC is globally reduced in *IDH1* wild-type (*IDH1* wt) GBM, regions with increased 5hmC associate with enhancers and actively transcribed genes [28]. Conflicting reports exist regarding the influence of *IDH1* mutation on global levels of 5hmC in gliomas, and to date no quantitative genome-wide locus-specific interrogation of 5hmC status in *IDH1* mutant (*IDH1* mt) tumors has been published to our knowledge [15, 16, 26, 28, 32, 33, 39, 48, 53, 67, 75, 78].

Taking advantage of oxidative bisulfite (OxBS) chemistry, 5mC can be differentiated from 5hmC. Herein, we examine a cohort of *IDH1* mt and *IDH1* wt HGG using BS and OxBS treatment, with downstream analysis on the Illumina Infinium^®^ MethylationEPIC BeadChip. We characterize 5hmC abundance and localization in *IDH1* mt tumors. We confirm 5hmC localization to enhancers in *IDH1* wt tumors and extend this finding to unique enhancer regions in *IDH1* mt tumors. The contribution of 5hmC to G-CIMP gene methylation is quantified. A significant association between gene body hydroxymethylation and gene expression is identified. Importantly, correlation between 5hmC abundance and gene expression for differentially expressed genes between *IDH1* mt and *IDH1* wt tumors is performed, demonstrating a significant association between increased 5hmC and genes upregulated in *IDH1* mt tumors.

## Materials and methods

### Study population

Fresh frozen human glioma samples (*n* = 21) were obtained from the University Health Network Brain Tumor Bank (Toronto, Canada). Samples were prospectively collected between 2002 and 2015 with written informed consent prior to surgery for use of tissues for research purposes. Local institutional review board approval for the work outlined in this manuscript was obtained prior to study initiation. DNA was extracted from specimens using the PureLink Genomic DNA kit (Invitrogen), and quantified using the Qubit dsDNA kit (Invitrogen). RNA was extracted using the Ambion WT kit (Ambion). RNA quality was assessed using Agilent 2100 Bioanalyzer.

### IDH1 and IDH2 mutation sequencing

*IDH1* and *IDH2* mutation status were confirmed by Sanger sequencing across the *IDH1* (*IDH1* R132) and *IDH2* (*IDH2* R172) mutation hotspots using the following published primer sets [21]:

- *IDH1*-Fwd 5′-GCACGGTCTTCAGAGAAGCCA-3′
- *IDH1*-Rev 5′-AGGGGATCCTATTGTGCAGCCAG-3′
- *IDH2*-Fwd 5′-AGCCCATCATCTGCAAAAAC-3′
- *IDH2*-Rev 5′-CTAGGCGAGGAGCTCCAGT-3′

For *IDH1*, genomic DNA was amplified using the following PCR cycling parameters: denaturation at 95 °C for 3 min, followed by 35 cycles of 95 °C for 30 s, 56 °C for 30 s and 72 °C at 45 s), with final extension at 72 °C for 10 min. For *IDH2*, an annealing temperature of 60 °C was used.

### Gene expression profiling

RNA was isolated from fresh frozen specimens as outlined above. Insufficient specimen was available from RNA extraction for gene expression profiling on one sample (sample 3921). RNA was assessed for purity by A260:A280 ratio, and RNA integrity using Agilent 2100 Bioanalyzer. Approximately 500 ng per sample was used as input for gene expression analysis by Affymetrix GeneChip™ Human Gene 2.0 ST array (Affymetrix). Arrays were processed by The Centre for Applied Genomics core facility (TCAG, Hospital for Sick Children, Toronto, Canada). Gene expression datasets were measured using the Affymetrix GeneChip Scanner 3000. Datasets were log_2_ transformed and quantile normalized. Robust Multiarray Average (RMA) background correction and additional quality checks were performed. Samples were combined into one data file before performing further analyses. To identify significant differences in gene expression between *IDH1* mt and *IDH1* wt tumors, *p* values were calculated using the function ANOVA in Partek Genomics Suite software (Partek, St. Louis USA). A log-rank test was performed to calculate *p* values. Genes with adjusted *p* values less than 0.05 and fold change above 2 were selected for further analyses. Gene set enrichment analysis (GSEA) of genes upregulated in *IDH1* mt and *IDH1* wt tumors was performed using the Broad Institute Molecular Signatures Database (version 6.0) [62].

### DNA hydroxymethylation and methylation profiling

Tumor DNA was isolated as described above. DNA for each sample (1 μg/sample) was processed in parallel using BS or OxBS conversion using the TrueMethyl^®^ Array kit (Cambridge Epigenetix), optimized for downstream analysis using the Illumina BeadChip array. The ssDNA concentrations of BS- and OxBS-converted aliquots were quantified with the Qubit ssDNA kit (Invitrogen). Methylcytosine and hydroxymethylcytosine profiling was performed as described by Stewart et al., adapted to the Illumina Infinium^®^ MethylationEPIC BeadChip [60]. 200 ng of BS- or OxBS-converted DNA was used as input for methylation profiling. Subsequent sample processing and Illumina BeadChip hybridization was performed by the Princess Margaret Genomics Centre core facility (Toronto, Canada).

### hMeDIP-Seq and hMeDIP-PCR validation

DNA (1 μg) from an expanded set of *IDH1* mt and *IDH1* wt gliomas was isolated as described above, and sheared by Bioruptor Pico sonicator (Diagenode). Sample clean-up was performed using AMPure XP beads (Agencourt), followed by end repair and adapter ligation using the NEBNext Ultra DNA Library Prep Kit (Illumina). Adaptor-ligated samples were size selected for 250–350 bp fragments using AMPure XP beads, denatured, and subject to overnight hydroxymethylation-dependent immunoprecipitation (hMeDIP) using an anti-5-hydroxymethylcytosine-specific antibody (Millipore, Clone HMC31). Prior to sonication and immunoprecipitation, all samples were spiked with an exogenous non-human 5hmC DNA control sequence (Zymo). PCR amplification and final library preparation for multiplexed sequencing of samples was performed using NEBnext Multiplex Oligos for Illumina as per the manufacturer’s instructions. Sequencing was performed on an Illumina HiSeq 2500 platform by The Centre for Applied Genomics (TCAG, Hospital for Sick Children, Toronto, Canada). Fastq files were checked for quality control using FastQC (v0.11.4) and adapters were trimmed using Cutadapt (v1.8). The resulting trimmed sequences were aligned to the UCSC GRCh37 genome using bwa (v0.7.8) and default parameters. Bam files were then imported into the MEDIPS (v1.20.1) package in R (v3.2.2), and their enrichment scores were determined to filter any samples that would fail the downstream analysis. The resulting samples that passed enrichment estimations (*IDH1* mt *n* = 24, *IDH1* wt *n* = 21) were run through MEDIPS using only unique reads to determine differentially hydroxymethylated regions across the genome between *IDH1* mt and *IDH1* wt, using the following parameters (uniq = 1e-3, extend = 300, shift = 1, ws = 100). Significant regions were then filtered by keeping regions that had an average of 3 or more reads, and whose log fold change was greater than ± 1, respectively. These regions were then intersected with gene annotations using bedtools (v2.21.0) to determine which genes have increased or decreased hydroxymethylation with *IDH1* mutation.

For hMeDIP-PCR, primers were designed flanking Illumina probe sites of interest, and quantitative real-time PCR performed to identify enrichment following immunoprecipitation. PCR results were normalized using PCR data for the spike-in Zymo control to control for IP efficiency based on the following formula: Enrichment = (Enrichment_target_)/(Enrichment_Zymo_) [37]. PCR primer sequences are listed in suppl. Table 1 (Online Resource 1).

### Data analysis

Loading and processing of methylation data were conducted using the Bioconductor package (version 3.3). Normalization and background correction of raw data IDAT files generated by Illumina Infinium^®^ MethylationEPIC BeadChips was performed using ssNoob in the Bioconductor package minfi (version 1.20.0) [2]. Quality analysis of samples was performed using principle component analysis (PCA). Probes corresponding to SNPs as well as those on sex chromosomes were removed, resulting in a total number of probes remaining for downstream analysis of 816,980. Pre-processed and normalized OxBS data were subtracted from BS data to generate ∆*β*-values representing 5hmC *β*-values per probe. Negative 5hmC *β*-values were adjusted to a value of 1 × 10^−7^ to approximate a zero value for 5hmC for those probes, as 5mC levels cannot exceed total mC and thus negative values represent technical artifact. Array annotation used for subsequent analyses was the IlluminaHumanMethylationEPICmanifest (version 0.3.0) available in Bioconductor. Calculated 5hmC *β*-values were compared with an alternate approach, employing the R package OxyBS to generate probe level 5hmC *β*-values, demonstrating strong concordance across all probe 5hmC *β*-values (suppl. Figure 1a, b, Online Resource 2) [24].

Top 1% 5hmC probes were identified by calculating the top 1% mean 5hmC *β*-values for all tumors in either the *IDH1* mt or *IDH1* wt cohorts [28]. Top 1% 5hmC probes identified by our approach demonstrated over 99.9% concordance with those identified using the OxyBS package (suppl. Figure 1c, d, Online Resource 2). Differentially methylated regions (DMRs) and DHMRs were identified through downstream analysis of minfi pre-processed/normalized data for 5mC or 5hmC using the Bumphunter algorithm (adjusted *p* value of 0.05, minimum number of probes = 7) in the Bioconductor package ChAMP [47]. Annotation of top 1% and DHMR probes with respect to genomic regions was performed using the package Goldmine in Bioconductor (https://github.com/jeffbhasin/goldmine) [5]. For hypergeometric probability calculations, the total number of protein-coding genes in the human genome was estimated at 20,345, based on GRCh37 (GENCODE v19). To generate volcano plots, mean 5hmC *β*-values for probes (all probes, non-overlapping top 1% probes, and DHMR probes) for *IDH1* mt and *IDH1* wt cohorts were calculated. Volcano plots were generated by calculating fold change and *p* value using *lmFit* and *ebayes* functions, demonstrating the degree of differential 5hmC across probes between *IDH1* cohorts. Cumulative density plots were generated using *ecdf* plot functions in the ggplot2 package.

Top 1% and DHMR probe enrichment for enhancer-related probes was performed by Cochran–Mantel–Haenszel test in Bioconductor. Annotations used to identify enhancer regions included the IlluminaHumanMethylationEPICmanifest. In addition, publically available H3K27Ac ChIP-seq enhancer data for three primary human GBM tumor propagating cell lines (MGG4, MGG6, MGG8), and H3K27Ac ChIP-seq enhancer and super enhancer data for normal human astrocytes were used as alternate annotations [23, 65]. Pathway enrichment analysis was performed using GeneAnalytics: pathways and gene ontology (biologic processes) performed using GeneAnalytics: GO-biologic process [4]. For enhancer probe pathway enrichment and GO-biologic process analyses, Illumina annotation was used to define enhancer-related probes.

For 5hmC-based consensus clustering, we used probes identified as being DHMRs that were also identified as being among the top 1% non-overlapping probes for *IDH1* mt or *IDH1* wt tumors (*n* = 321). Unsupervised consensus clustering was performed to define the number of clusters in an unbiased fashion, using the Bioconductor package ConsensusClusterPlus [56]. Pearson correlation was used for the distance metric, and Ward for the linkage algorithm, with 1000 re-sampling steps performed (epsilon = 0.8).

Sample clustering based on 5mC (Pearson dissimilarity, ward method) was performed using OxBS-data *β*-values for all probes for 50 genes identified as significantly hypermethylated in G-CIMP gliomas [52]. For the 5hmC heatmap, probe and tumor samples were sorted in a supervised manner based on probe and tumor location as per the 5mC heatmap, to allow visualization of 5hmC contribution to overall methylation within the *IDH1* mt cohort across the 50 G-CIMP gene probes. Spearman correlation coefficients between 5hmC/5mC and gene expression, as well as between 5hmC and 5mC *β*-values, were calculated using the *cor* test function in Bioconductor.

## Results

### IDH1 mutation status and differential gene expression between IDH1 mt and wt gliomas

DNA and mRNA were isolated from 21 fresh frozen human high-grade glioma samples (*IDH1* mt *n* = 12, *IDH1* wt *n* = 9). Sample characteristics and patient demographics are summarized in Table 1. *IDH1* mutation was confirmed by Sanger sequencing, with 11/21 samples harboring *IDH1* R132H mutations and 1/21 the *IDH1* R132C variant (suppl. Figure 2, Online Resource 3). No *IDH2* (R172) mutations were identified. The difference in mean age between *IDH1* mt (40.6 years) and *IDH1* wt (58.8 years) was statistically significant (*p* value = 0.0009), as expected with *IDH1* mutation typically identified in younger cohorts.

**Table 1:** Tumor sample characteristics

	*IDH1* mutant cohort	*IDH1* wild-type cohort
Pathology (WHO grade)		
Anaplastic astrocytoma (III)	6 (50%)	0 (0%)
High-grade glioma (III/IV)	3 (25%)	0 (0%)
GBM (IV)	3 (25%)	9 (100%)
IDH1 mutation status		
R132H	11 (91.7%)	0 (0%)
R132C	1 (8.3%)	0 (0%)
Age at diagnosis		
Median	40.6	58.8
Range	26–54	37–77
Sex		
Male	7 (58.3%)	5 (55.6%)
Female	5 (41.7%)	4 (44.4%)

Affymetrix HumanGene 2.0 ST arrays were used to profile gene expression, identifying genes with a minimum twofold differential expression between *IDH1* mt and *IDH1* wt tumors (suppl. Table 2, Online Resource 4). In total, 673 genes were upregulated in *IDH1* mt tumors. Gene set enrichment analysis (GSEA) of these genes identified significant overlap with the Verhaak GBM Proneural gene set (*p* value = 8.59 × 10^−90^, *q* value = 2.92 × 10^−86^) [46, 62].

### Quantification and distribution of 5hmC and 5mC in IDH1 mt versus wt gliomas

The mean probe 5hmC *β*-value for *IDH1* mt tumors was 0.046 (standard deviation 0.013) versus 0.037 (standard deviation 0.009) for *IDH1* wt tumors. *IDH1* mt tumors demonstrated a non-significant trend toward higher mean 5hmC (*p* value = 0.0916). Overall 5hmC abundance did not strongly parallel 5mC abundance, as Spearman correlation (*r*_s_) between mean 5hmC versus 5mC across tumor samples failed to identify a significant correlation (*r*_s_ = 0.308, *p* value = 0.1747). While patient age differed significantly between *IDH1* mt and *IDH1* wt cohorts, no correlation was evident between patient age and mean 5hmC *β*-values (*r*_s_ = − 0.02, *p* value = 0.92).

In contrast to 5mC, cumulative density plots of 5hmC *β*-values showed near-zero values for most probes, with 5hmC *β*-values greater than 0.1 accumulating in the 90th percentile for both *IDH1* cohorts (suppl. Figure 3a, b, Online Resource 5). Stratifying probes by CpG island feature, 5mC targeted CpG island shelves, followed by shores, and lastly CpG islands in *IDH1* mt and *IDH1* wt tumors (suppl. Figure 3c, d, Online Resource 5). In contrast, 5hmC targeted CpG island shores and shelves to a similar degree, and more so than CpG islands (suppl. Figure 3e, f, Online Resource 5). Similar patterns have been previously demonstrated in *IDH1* wt GBM [28].

### Assessment of top 1% and differentially hydroxymethylated region probes

To examine 5hmC further, we performed two parallel analyses: characterization of probes demonstrating the highest 5hmC abundance, and characterization of DHMRs between *IDH1* cohorts. To examine regions of high 5hmC abundance, we identified probes within the top 1% mean 5hmC *β*-values for *IDH1* mt and *IDH1* wt cohorts (suppl. Table 3, Online Resource 6) [28, 41]. Top 1% 5mC probes for *IDH1* mt and *IDH1* wt subgroups were also identified (suppl. Table 3, Online Resource 6). For top 1% 5hmC probes, mean *β*-value for *IDH1* mt tumors was 0.189 (standard deviation 0.051) versus 0.157 (standard deviation 0.042) for *IDH1* wt tumors. While the mean *β*-value for the top 1% 5hmC probes was higher in *IDH1* mt tumors, this difference was not significant (*p* value = 0.1424). At the individual tumor level, mean 5hmC *β*-values for top 1% probes ranged from 0.139 to 0.301 for *IDH1* mt tumors, and 0.082 to 0.211 for *IDH1* wt tumors. Examining mean 5hmC *β*-values with respect to patient age, no significant correlation was evident (*r*_s_ = − 0.20, *p* value = 0.38). In relation to CpG island features, probes with the greatest 5hmC abundance targeted Open Sea regions, followed by CpG island shores in both *IDH1* mt and *IDH1* wt tumors (suppl. Figure 4a, Online Resource 7). This was in contrast to top 1% 5mC probes which targeted Open Sea regions followed by CpG islands (suppl. Figure 4b, Online Resource 7). Stratified by gene region, top 1% 5hmC probes predominantly annotated to introns, whereas top 1% 5mC probes targeted promoters (suppl. Figure 4c, d, Online Resource 7). Taken together, this suggests regions with greater 5hmC abundance may exert epigenetic influence within gene body regions or at distal regulatory sites such as enhancer regions, in contrast to the promoter CpG island targeting evidenced by 5mC.

Absolute 5hmC values were an order of magnitude lower than 5mC. As regions with lower 5hmC *β*-values could conceivably contribute to epigenetic regulation, limiting our analysis to probes within the top 1% 5hmC *β*-values might exclude regions of significance. As such, we used a second approach identifying DHMRs (adjusted *p* value threshold of 0.05) between *IDH1* mt and *IDH1* wt tumors using the Bioconductor package ChAMP, irrespective of absolute *β*-values [47]. DHMRs consisted of 15,279 probes targeting 1850 annotated genes (suppl. Table 4, Online Resource 8). With respect to CpG island features, DHMR probes targeted CpG islands, followed by CpG island shores, and Open Sea regions (suppl. Figure 4e, Online Resource 7). Annotated by gene region, DHMR probes associated mainly with promoter regions (suppl. Figure 4f, Online Resource 7).

**Fig. 1:**
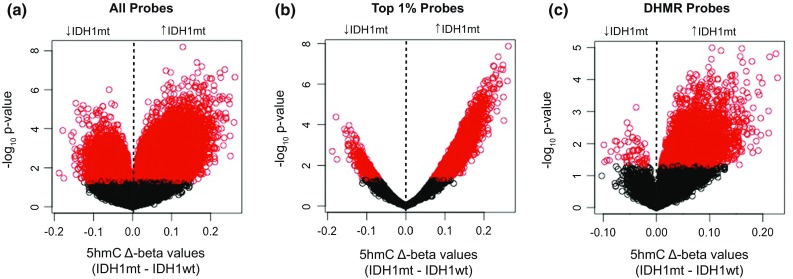
Genome-wide enrichment of 5hmC in *IDH1* mutant versus *IDH1* wild-type tumors. Volcano plots of 5hmC ∆*β*-value (*IDH1* mt–*IDH1* wt) versus – log_10_(*p* value) using mean 5hmC *β*-value per probe within each *IDH1* cohort, for **a** all probes, **b** top 1% probes, and **c** DHMR probes. ∆*β*-values with *p* value < 0.05 are shown in red. Compared with all EPIC BeadChip probes, probes among the top 1% 5hmC abundance and differentially hydroxymethylated probes demonstrate an asymmetric distribution with increased 5hmC in *IDH1* mt tumors

Plotting the difference between mean *IDH1* mt 5hmC *β*-value minus mean *IDH1* wt 5hmC *β*-value per probe (5hmC ∆*β*-value) against – log_10_(*p* value), we noted slightly greater 5hmC in *IDH1* mt tumors (Fig. 1a). Comparing top 1% 5hmC probes or DHMR probes, a greater proportion of probes demonstrated increased 5hmC in the *IDH1* mt cohort (Fig. 1b, c), suggesting that the bulk of differential hydroxymethylation seen between *IDH1* mt and *IDH1* wt tumors is secondary to *greater* hydroxymethylation in the *IDH1* mt cohort.

### Pathway enrichment and gene ontology analyses for top 1% and DHMR probes

Top 1% 5hmC probes for *IDH1* mt and *IDH1* wt tumors only partially overlapped (suppl. Figure 5a, Online Resource 9). Pathway enrichment analysis of top 1% 5hmC gene targets identified common and unique pathways between *IDH1* cohorts, many of which have been implicated in GBM pathogenesis (suppl. Figure 5b, Online Resource 9). Common pathways included pathways in cancer, *ERK* signaling, and dopamine D2 receptor transactivation of *EGFR*. *HGF* signaling, *TGFβ* receptor signaling, and apoptotic pathways were unique to *IDH1* mt tumors. Pathways exclusive to *IDH1* wt tumors included Wnt/Hedgehog/Notch and *RAS* signaling. Pathway analysis for DHMR probes identified *ERK* signaling, and Wnt/Hedgehog/Notch signaling in common with top 1% probes. In addition, *MYC*-mediated transcriptional repression and human embryonic stem cell pluripotency were identified (suppl. Figure 5c, Online Resource 9). Analysis of gene ontology for enriched biological processes identified response to hypoxia, nervous system development and cell differentiation among the most significant processes for DHMR probes (suppl. Table 5, Online Resource 10).

### Enhancer and super-enhancer targeting by 5hmC in IDH1 mt and IDH1 wt gliomas

**Fig. 2:**
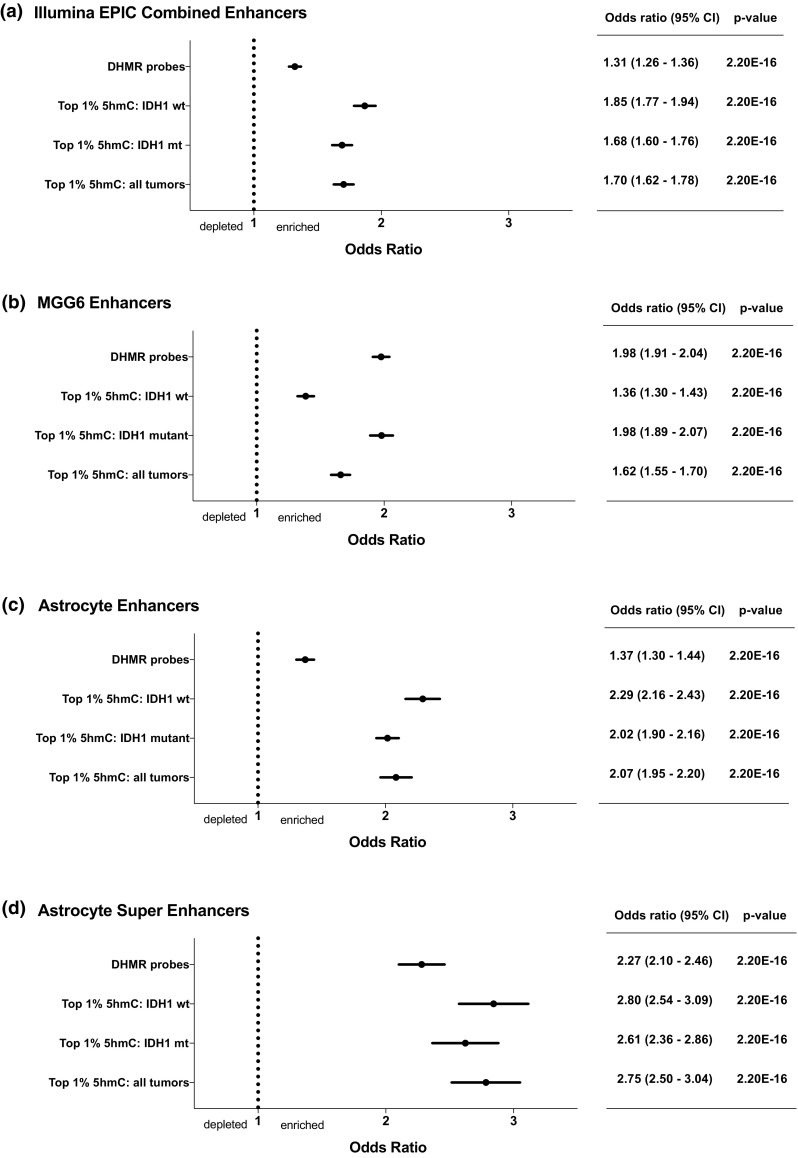
5hmC targets enhancer and super-enhancer regions in *IDH1* mutant and *IDH1* wild-type high-grade gliomas. Forest plots depicting odds ratios (OR), 95% confidence intervals (95% CI) and associated *p* values for enriched enhancer targeting by top 1% 5hmC probes for all tumors combined, *IDH1* mt and *IDH1* wt top 1% probes individually, and for DHMR probes. Multiple enhancer annotations were used including the **a** Illumina EPIC BeadChip annotation, as well as **b** H3K27Ac ChIP-seq of human primary GBM tumor propagating cell line MGG6, and **c** normal human astrocytes. **d** Enriched super-enhancer targeting, based on super-enhancer regions identified by H3K27Ac ChIP-seq of normal human astrocytes

As probes demonstrating the greatest 5hmC localized predominantly in Open Sea regions, we sought to determine if such probes target distal regulatory elements such as enhancer regions. 5hmC enrichment at enhancer and super-enhancer regions has been reported during development, within normal brain, and recently among a cohort of *IDH1* wt GBMs [28, 57, 73]. We sought to confirm this finding and determine if it extended to *IDH1* mt tumors. Multiple enhancer annotations were used for this analysis. Based on the Illumina Infinium^®^ MethylationEPIC BeadChip annotation, significant association with enhancer regions was demonstrated for DHMR and top 1% 5hmC probes (Fig. 2a). Histone H3 lysine 27 acetylation (H3K27Ac) marks active enhancers, and serves as a surrogate for identifying enhancers genome wide [23]. Publically available H3K27Ac ChIP-seq data for primary human GBM cell lines were used to annotate DHMR and top 1% 5hmC probes, again identifying significant enhancer targeting (Fig. 2b) [65]. Data for GBM cell line MGG6 annotation are shown, with data for MGG4 and MGG8 lines in suppl. Figure 6 (Online Resource 11). Publically available H3K27Ac ChIP-seq data for normal human astrocyte enhancers and super-enhancers were also used to annotate DHMR and top 1% 5hmC probes. Enrichment for probes targeting enhancer regions was seen for DHMR and top 1% 5hmC probes, with even greater enrichment observed for super-enhancers (Fig. 2c, d).

**Fig. 3:**
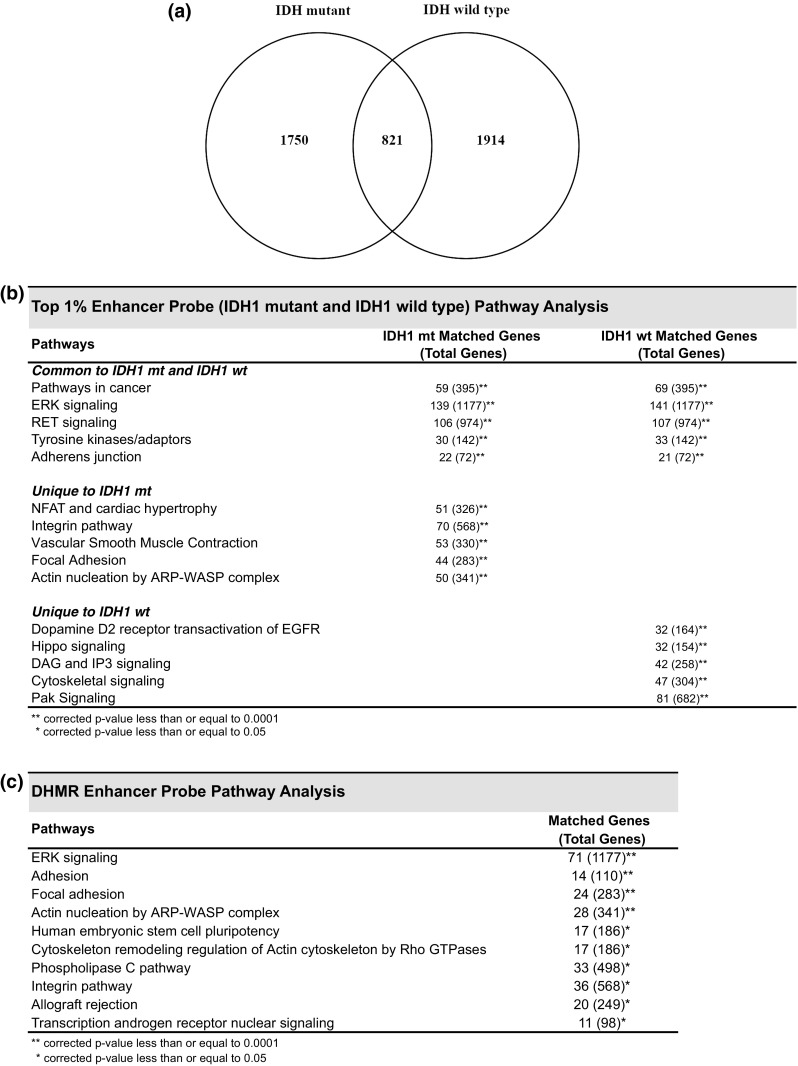
Pathway analysis for top 1% and DHMR enhancer probes. **a** Venn diagram depicting < 1/3 overlap between *IDH1* mt and *IDH1* wt top 1% 5hmC enhancer probes. **b** Pathway analysis for top 1% 5hmC enhancer-related probe targets. Unique and common pathways identified among the top 10 pathways for each of *IDH1* mt and *IDH1* wt tumors are listed, along with the number of matched genes observed for each pathway. **c** Pathway analysis for DHMR enhancer probes

While enhancer targeting by top 1% 5hmC probes was a feature of both *IDH1* mt and *IDH1* wt tumors, enhancer-related probes demonstrated less than 1/3 overlap between *IDH1* cohorts (Fig. 3a). Common pathways identified for top 1% enhancer probes included pathways in cancer, *ERK* signaling, and tyrosine kinases/adaptors (Fig. 3b). Pathways unique to *IDH1* mt top 1% enhancer probes included *NFAT* and cardiac hypertrophy, integrin pathway, focal adhesion, and actin nucleation by *ARP*–*WASP* complex. Pathways exclusive to top 1% *IDH1* wt enhancer probes included dopamine D2 receptor transactivation of *EGFR*, Hippo signaling, DAG and IP3 signaling, cytoskeletal signaling, and Pak signaling. Pathway analysis based on DHMR enhancer probes also identified *ERK* signaling, focal adhesion, integrin pathway, and actin nucleation by *ARP*–*WASP* complex (Fig. 3c). Among additional enriched pathways in DHMR enhancer probes was human embryonic stem cell pluripotency. Analysis of gene ontology for enriched biological processes identified response to hypoxia as the most significant biologic process for DHMR enhancer probes (suppl. Table 6, Online Resource 12).

### 5hmC-based consensus clustering identifies co-segregation for a subset of IDH1 mutant and wild-type tumors

Consensus clustering was performed based on 5hmC *β*-values for select probes in common between DHMRs and top 1% probes (*n* = 321). In total, we identified three robust clusters (suppl. Figures 7, 8, Online Resources 13, 14). Cluster 1 and cluster 3 consisted solely of *IDH1* mt and *IDH1* wt tumors, respectively. Cluster 2 was mixed, with 2 *IDH1* mt and 3 *IDH1* wt tumors. Cluster membership did not correlate with patient age or tumor grade. Gene expression analysis identified primarily genes differentially expressed between *IDH1* mt and *IDH1* wt when comparing clusters 1 and 3, while no significant differentially expressed genes were identified comparing the mixed cluster (cluster 2) with either the pure *IDH1* mt or wt clusters (suppl. Table 7, Online Resource 15). These data imply that, while *IDH1* status represents one factor altering 5hmC profile, additional mechanisms must influence 5hmC patterns resulting in a mixed *IDH1* mt and wt population cluster.

### 5hmC contributes to overall methylation of G-CIMP signature genes

**Fig. 4:**
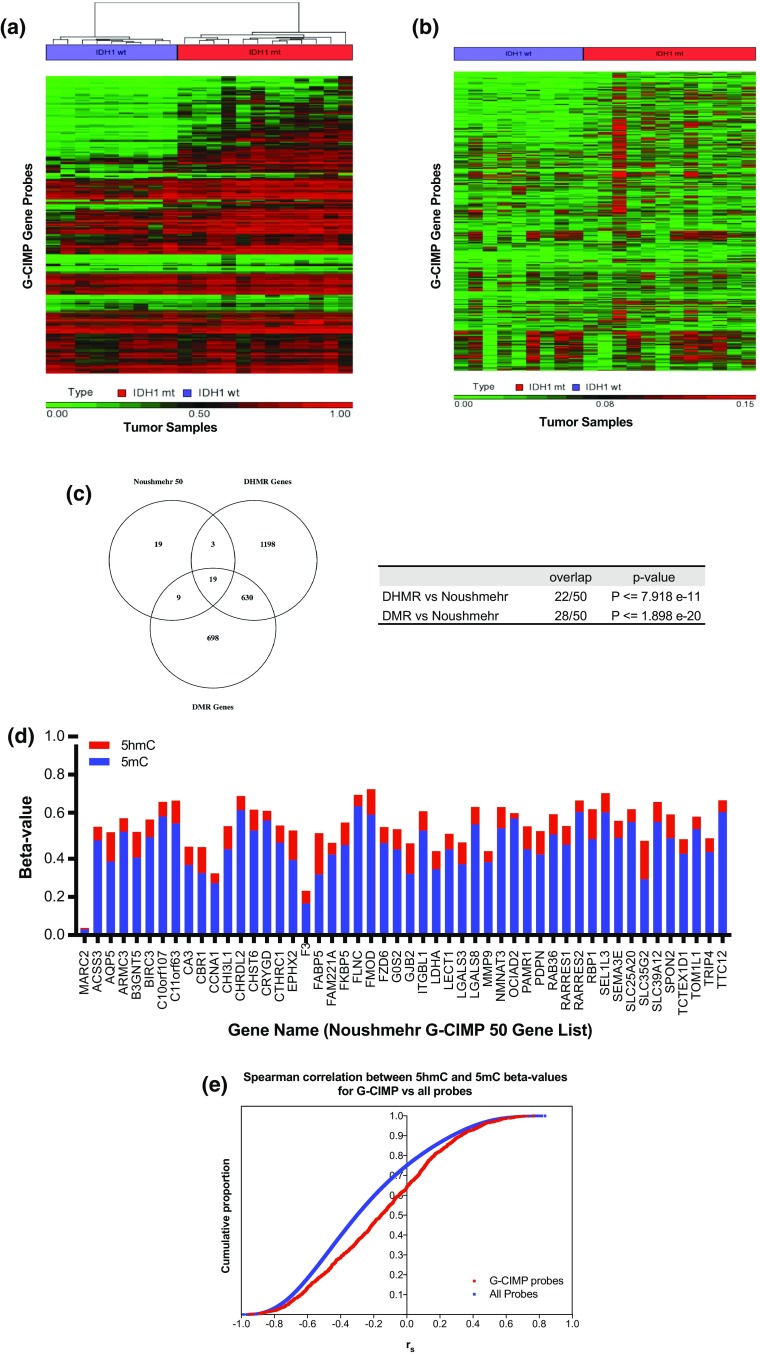
5hmC contributes to overall cytosine methylation of G-CIMP genes in *IDH1* mutant tumors. **a** Hierarchical clustering and associated heatmap based on OxBS-derived 5mC *β*-values for all probes targeting each of 50 G-CIMP genes. Clustering based on 5mC profiles separates *IDH1* mt from *IDH1* wt tumors, and demonstrates increased cytosine methylation typical of G-CIMP in *IDH1* mt tumors. **b** Heatmap depicting 5hmC *β*-values for the G-CIMP 50 gene list probes organized as per the 5mC heatmap in Fig. 4a. Variable contribution of 5hmC to overall methylation for G-CIMP genes among *IDH1* mt tumors is depicted. **c** Venn diagram demonstrating the overlap between the G-CIMP 50 gene list and gene targets of DHMRs and DMRs. Hypergeometric distribution *p* values signifying significant overlap. **d** Relative contribution of 5hmC and 5mC to overall cytosine methylation of G-CIMP genes, based on mean 5hmC and 5mC *β*-values per gene. Example shown is for *IDH1* mt sample 1730. **e** Cumulative density plot of Spearman correlation coefficients (*r*_s_) between 5hmC and 5mC *β*-values for all G-CIMP gene probes (red), compared to similar correlation across all Illumina Infinium^®^ MethylationEPIC BeadChip probes (blue). The proportion of probes demonstrating negative *r*_s_ values for G-CIMP gene probes is significantly lower than that seen for all probes (*z* score = 9.565, *p* value = 0)

OxBS-derived 5mC *β*-values for 50 G-CIMP signature gene probes (Noushmehr 50 genes) were used to perform unsupervised hierarchical clustering of tumors in our dataset [52]. Clustering based purely on 5mC data clearly separated *IDH1* wt from *IDH1* mt tumors (Fig. 4a). 5hmC *β*-values for G-CIMP gene probes were depicted as a heatmap in Fig. 4b, organized as per sample and gene location in Fig. 4a. For a subset of *IDH1* mt tumors, 5hmC contributed to overall methylation targeting G-CIMP signature genes. Despite this, clustering based on 5mC alone was sufficient to segregate tumors by G-CIMP status. Of G-CIMP signature genes, 28/50 were differentially methylated in our dataset (Fig. 4c). This overlap was statistically significant (hypergeometric probability *p* value ≤ 1.898 × 10^−20^). In addition, 22/50 genes were differentially hydroxymethylated (hypergeometric probability *p* value ≤ 7.918 × 10^−11^).

To examine the contribution of 5hmC to overall methylation for G-CIMP genes quantitatively, mean 5hmC *β*-value per gene was expressed as a percentage of the mean total methylation (5mC + 5hmC) (suppl. Table 8, Online Resource 16). Average gene-level 5mC and 5hmC data were used to minimize bias related to differential 5mC or 5hmC that might have affected only a subset of probes for any particular gene. Examining genes with methylation *β*-values of ≥ 0.3, the overall contribution of 5hmC to total methylation across the 50-gene list ranged from 7.00 to 17.75% per tumor in *IDH1* mt tumors. At the individual gene level, percent contribution of 5hmC to overall methylation ranged as high as 40.97%. As an example, Fig. 4d depicts the relative contributions of 5hmC and 5mC to overall methylation for 50 G-CIMP genes in one *IDH1* mt tumor (sample 1730, GBM). This GBM specimen demonstrated the greatest 5hmC levels compared to others in the *IDH1* mt cohort. This sample was unique among the *IDH1* mt tumors as it represented a GBM with gliosarcoma phenotype. It is unknown at present whether elevated 5hmC is a prominent feature of this GBM pathologic subtype.

Hypermethylation of genes in G-CIMP tumors has been mechanistically linked to *IDH1* mutation, with the oncometabolite 2-HG inhibiting TET enzyme-mediated demethylation [68]. As such, a strong negative correlation demonstrating 5mC accumulation and 5hmC loss at CpG sites is expected—in particular, for sites representative of G-CIMP. Interestingly, correlation between 5hmC and 5mC for G-CIMP gene probes demonstrated significantly fewer probes with a negative Spearman’s correlation compared to all probes across the MethylationEPIC BeadChip (Fig. 4e). For G-CIMP gene probes, a negative correlation between 5mC and 5hmC was seen in 63.98%, compared to 75.01% for all probes. Our data for all probes are in line with previous reports demonstrating 80% negative correlation between 5hmC and 5mC in *IDH1* wt GBMs [28]. The observed reduced difference in proportion of probes with negative correlations between 5mC and 5hmC for G-CIMP gene probes in our data was highly significant (*z* score = 9.565, *p* value = 0). These findings suggest that altered 5mC and 5hmC levels in *IDH1* mt/G-CIMP gliomas may be due to additional factors aside from impaired TET-mediated conversion of 5mC to 5hmC alone. It is also possible that TET inhibition not only impacts 5mC abundance but also 5hmC due to altered downstream oxidation of 5hmC to 5fC.

### Gene body 5hmC correlates with highly expressed genes

To examine the association between 5hmC and gene expression, top 1% 5hmC probes for *IDH1* mt and *IDH1* wt tumors were correlated with gene expression for the top 20% most highly expressed genes based on our Affymetrix expression data (suppl. Table 9, Online Resource 17). For both *IDH1* cohorts, increased 5hmC was significantly associated with genes expressed in the top 20th percentile (*p* < 0.0001), with an OR of 2.26 (95% CI 2.16–2.36) for *IDH1* mt and an OR of 1.93 (95% CI 1.84–2.02) for *IDH1* wt tumors. Stratified by genomic region, top 1% 5hmC probes surrounding transcription start sites (TSS200/1st exon probes) were associated with reduced gene expression, whereas probes targeting gene body regions were most significantly associated with elevated gene expression for both *IDH1* cohorts (suppl. Table 9, Online Resource 17). Hydroxymethylated *IDH1* mt gene body probes demonstrated an OR of 2.27 (95% CI 2.14–2.42) for association with genes expressed in the top 20th percentile, while *IDH1* wt gene body probes demonstrated an OR of 2.08 (95% CI 1.95–2.21). This association between gene body hydroxymethylation with increased expression and TSS200/1st exon hydroxymethylation with reduced expression is depicted in suppl. Figure 9a, b (Online Resource 18). To assess the influence of 5mC on gene expression, we assessed probes with mean 5mC *β*-values ≥ 0.7 as well as those with *β*-values ≤ 0.3 with respect to their association with genes in either the top or bottom 20th percentiles for gene expression. 5mC probes with *β*-values ≥ 0.7 targeting TSS200/1st exon regions were significantly associated with reduced gene expression, with an OR of 5.22 (95% CI 4.92–5.55) for *IDH1* mt and an OR of 3.96 (95% CI 3.69–4.26) for *IDH1* wt tumors (suppl. Table 10, Online Resource 19). In contrast, 5mC probes with *β*-values ≤ 0.3 targeting TSS200/1st exon regions were significantly associated with genes expressed in the top 20th percentile, with an OR of 5.81 (95% CI 5.53–6.10) for *IDH1* mt and an OR of 5.84 (95% CI 5.52–6.17) for *IDH1* wt cohorts. 5mC probes targeting gene body regions failed to demonstrate a strong or consistent association with highly expressed genes. The association between genes expressed in the top 20th percentile and probes targeting gene bodies with *β*-values ≥ 0.7 demonstrated an OR of 0.84 (95% CI 0.82–0.85) for *IDH1* mt tumors, and an OR of 1.12 (95% CI 1.11–1.14) for *IDH1* wt tumors (suppl. Table 10, Online Resource 19).

### 5hmC localizes to highly expressed genes in IDH1 mt tumors

To examine the influence of 5hmC on differential gene expression between *IDH1* mt and *IDH1* wt tumors at a gene-specific level, *β*-values for top 1% 5hmC and DHMR probes were correlated with differentially expressed genes between *IDH1* subgroups. Analysis was restricted to probes showing strong correlations with gene expression (Spearman correlation coefficient values of |*r*_*s*_| ≥ 0.5). In total, 85 probes (75 genes) and 406 probes (128 genes) met these criteria among top 1% and DHMR probes, respectively (suppl. Table 11, Online Resource 20). Among probes demonstrating a significant Spearman correlation with gene expression, the majority targeted gene body regions (80.0% for top 1% probes, 59.1% for DHMR probes).

**Fig. 5:**
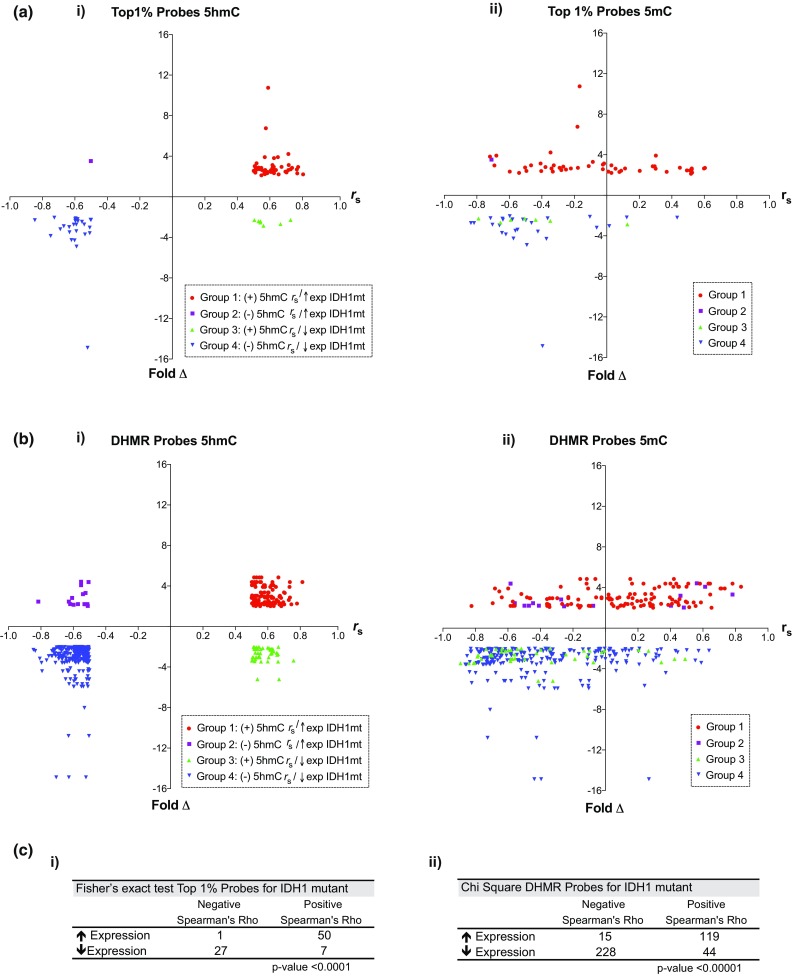
5hmC strongly correlates with genes highly expressed in *IDH1* mutant tumors. **a-i** Spearman correlation coefficients (*r*_s_) between 5hmC *β*-values for top 1% probes versus gene expression for genes differentially expressed between *IDH1* cohorts were determined. Probes with |*r*_s_| ≥ 0.5 are graphed as a scatter plot showing *r*_s_ on the *x*-axis against fold change (Fold ∆, *IDH1* mt vs *IDH1* wt) on the *y*-axis. Probes are divided into four groups based on 5hmC Spearman correlation (+ or −) and associated gene expression in *IDH1* mt tumors (increased or decreased). Group 1 represents probes with positive *r*_s_ targeting genes upregulated in *IDH1* mt tumors. Group 2 represents probes with negative *r*_s_ targeting genes upregulated in *IDH1* mt tumors. Group 3 represents probes with positive *r*_s_ targeting genes downregulated in *IDH1* mt tumors. Group 4 represents probes with negative *r*_s_ targeting genes downregulated in *IDH1* mt tumors. **a-ii** Spearman correlation coefficients for 5mC probe *β*-values versus gene expression for the probes depicted in Fig. 5a-i were determined and graphed as a scatter plot. Group definitions are based on *5hmC* Spearman correlations with gene expression as in Fig. 5a-i. Group 1 probes (+5hmC correlation and increased expression in *IDH1* mt) demonstrate variable positive and negative correlation between 5mC levels and gene expression. Group 2–4 probes demonstrate predominantly negative correlation between 5mC and associated gene expression. **b-i** and **b-ii** Scatter plots as described in Fig. 5a, based on DHMR probes. Groups 1–4 as defined in Fig. 5a-i are highlighted, demonstrating similar trends with respect to 5hmC and 5mC Spearman correlation coefficients with gene expression versus fold change in gene expression between *IDH1* mt and *IDH1* wt subgroups. **c** Summary of probe counts for Group 1–4 probes, and Fisher’s exact test (for top 1% probes) or Chi-square statistic (for DHMR probes) demonstrating significant over-representation of 5hmC probes with positive Spearman correlation with genes upregulated in *IDH1* mt tumors, for both top 1% (**c-i**) and DHMR (**c-ii**) probe sets

To assess the relationship between 5hmC and gene expression further, probes were divided into four groups based on whether the Spearman correlation coefficient was positive or negative, and whether the gene target demonstrated increased or decreased expression in the *IDH1* mt cohort. For top 1% 5hmC probes, genes demonstrating increased expression in *IDH1* mt tumors predominantly demonstrated a positive correlation between 5hmC and gene expression (Fig. 5a-i, Group 1). Among *IDH1* mt upregulated genes, only a single probe demonstrated a negative correlation between 5hmC and gene expression (Fig. 5a-i, Group 2). Group 3 probes exhibited a positive correlation between 5hmC and gene expression for genes with reduced expression in *IDH1* mt tumors (Fig. 5a-i, Group 3). Lastly, Group 4 probes demonstrated negative correlations between 5hmC and gene expression for genes with decreased expression in *IDH1* mt tumors (Fig. 5a-i, Group 4).

To exclude underlying influence of 5mC on gene expression for this subset of probes, correlation between 5mC *β*-values and gene expression was performed. This analysis did not identify a consistent trend between 5mC and gene expression for Group 1 probes (Fig. 5a-ii, Group 1), whereas Group 2–4 probe Spearman correlations between 5mC and gene expression were largely negative (Fig. 5a-ii, Groups 2–4).

For Group 1, all probes (50/50) demonstrated higher mean 5hmC *β*-values in *IDH1* mt tumors, with 84% of probes targeting transcribed gene body regions, suggesting that increased 5hmC facilitates increased expression (suppl. Table 11, Online Resource 20). For the single Group 2 probe, the mean 5mC *β*-value was lower among *IDH1* mt tumors. Increased expression of this gene target in *IDH1* mt tumors may be related to lower 5mC facilitating transcription. For all Group 3 probes, discordant 5hmC and 5mC correlation was observed, with increased 5hmC associated with increased gene expression, and increased 5mC associated with decreased gene expression. In this group, mean 5hmC *β*-values were higher among *IDH1* wt tumors for all probes (7/7), and mean 5mC *β*-values higher among *IDH1* mt for all probes (7/7). Therefore, increased expression of Group 3 genes seen in *IDH1* wt tumors may be facilitated by either a positive effect on transcription by 5hmC in *IDH1* wt tumors or conversely an inhibitory influence of greater 5mC in *IDH1* mt tumors. For Group 4 probes, the negative association between 5hmC and gene expression was largely concordant with a negative association between 5mC and gene expression. In this group, 26/27 probes had greater mean 5mC *β*-values in *IDH1* mt tumors and, therefore, reduced expression in *IDH1* mutant tumors for Group 4 genes might be a reflection of the silencing effect of 5mC.

Examining DHMR 5hmC probes, a similar correlation between 5hmC and gene expression was observed for genes overexpressed in *IDH1* mt tumors (Fig. 5b-i, Group 1). In comparison to 5hmC, 5mC correlations with gene expression for DHMR Group 1 probes covered a broad spectrum between negative and positive values, as was observed in our top 1% probe analysis (Fig. 5b-ii, Group 1). Similar to results seen with top 1% probes, 5mC Spearman correlations for DHMR Group 2–4 probes were predominantly negative (Fig. 5b-ii, Group 2–4).

As with top 1% Group 1 probes, the majority of DHMR Group 1 probes (118/119) demonstrated higher mean 5hmC *β*-values in *IDH1* mt tumors, with 75.6% targeting transcribed gene body regions (suppl. Table 11, Online Resource 20). Increased 5hmC may facilitate increased expression of these genes in the *IDH1* mt cohort. In contrast, DHMR Group 2 probes predominantly demonstrated mean 5hmC *β*-values higher among *IDH1* wt tumors (14/15). As with top 1% probes, nearly all DHMR Group 3 probes (43/44) demonstrated mean 5hmC *β*-values higher in *IDH1* wt tumors. Lastly, among DHMR Group 4 probes, 227/228 had higher mean 5hmC *β*-values in *IDH1* mt tumors, and the majority (213/228) also demonstrated higher mean 5mC *β*-values among *IDH1* mt tumors. As such, reduced expression of Group 4 genes may be mediated epigenetically by 5mC-mediated silencing. The differential distribution for top 1% and DHMR probes based on 5hmC Spearman correlations with gene expression were highly statistically significant (Fig. 5c).

Among gene targets of probes demonstrating a positive correlation between 5hmC and increased gene expression in *IDH1* mt tumors (Group 1) were several genes implicated in glioma pathogenesis, including leucine-rich repeat containing G protein-coupled receptor 5 (*LGR5*). *LGR5* has been implicated in promoting the glioma stem cell phenotype [3, 22, 44, 45, 49, 72, 79]. As seen in suppl. Figure 10 (Online Resource 21) our data demonstrated a significant positive Spearman correlation between *LGR5* gene expression and 5hmC *β*-values (*r*_s_ = 0.703, *p* value = 0.00055), and a non-significant correlation with 5mC (*r*_s_ = 0.203, *p* value = 0.39167). Average log_2_ expression for *LGR5* was 7.08 (standard deviation 0.08) and 5.94 (standard deviation 0.93) for *IDH1* mt and IDH1 wt tumors, respectively, representing a 2.41-fold increased expression in *IDH1* mt tumors (*p* value = 0.0065). Average 5hmC *β*-value for this probe (cg23900866) in *IDH1* mt tumors was 0.18 (17.75%, standard deviation 0.139) and in *IDH1* wt tumors was 0.03 (3.44%, standard deviation 0.054), representing a statistically significant difference (*p* value = 0.0097).

### Validation of 5hmC targets by hMeDIP

Verification of 5hmC gene targets was performed using an hMeDIP-Seq and hMeDIP-PCR approach on an overlapping, expanded set of *IDH1* mt (*n* = 24) and *IDH1* wt (*n* = 21) tumors (suppl. Table 12, Online Resource 22). Of samples assessed by Illumina MethylationEPIC arrays, 17/21 passed QC and were included in hMeDIP-Seq analysis. Comparing genes identified as differentially hydroxymethylated between *IDH1* mt and *IDH1* wt tumors by hMeDIP-Seq (*n* = 2379) versus DHMR genes identified by Illumina MethylationEPIC BeadChip using ChAMP (*n* = 1850), we found 267 common differentially hydroxymethylated targets. This extent of common targets between the two modalities was statistically significant (hypergeometric probability *p* ≤ 1.03 × 10^−4^). Using quantitative hMeDIP-PCR on samples where sufficient DNA was available, we confirmed increased 5hmC among an expanded *IDH1* mt tumor cohort (*n* = 25). Enrichment following hMeDIP was confirmed for multiple gene candidates including *LGR5*, *WDR11*-*AS1*, *CRTAC1*, *GRID1*, and *FAM155A* among the *IDH1* mt tumors (suppl. Figure 11, Online Resource 23).

## Discussion

*IDH1* mutation is observed frequently in LGG and a subset of HGG, resulting in a gain of function leading to 2-HG production [1]. 2-HG inhibits α-ketoglutarate-dependent enzymes, including TET cytosine demethylases [75]. As such, dysregulation of both 5mC and 5hmC homeostasis would be expected in *IDH1* mt tumors. In recent years, investigation into the role aberrant 5hmC plays in gliomagenesis has begun. However, to our knowledge, no quantitative locus-specific genome-wide characterization of 5hmC in *IDH1* mt gliomas has been published.

Here, we profiled 5hmC in *IDH1* mt and *IDH1* wt HGGs through parallel processing of BS- and OxBS-converted samples using the Illumina Infinium^®^ MethylationEPIC BeadChip. We characterized the abundance and distribution of 5hmC in *IDH1* mt versus *IDH1* wt tumors, assessed the contribution of 5hmC to G-CIMP gene methylation, and examined the correlation between 5hmC and gene expression including genes differentially expressed between *IDH1* cohorts. One limitation of this study relates to the small cohort sizes examined, and the heterogeneous nature of pathologic diagnoses in the *IDH1* mt subgroup. It has been shown, however, that histopathology may fail to reliably distinguish between WHO grade III and IV tumors, and that *IDH1* mutation status is a stronger prognostic factor compared to patient age or pathologic diagnostic category [20]. As such, WHO grade III and IV *IDH1* mt tumors were assessed together as a single cohort.

In our study, overall levels of hydroxymethylation did not differ significantly between *IDH1* cohorts, with an *IDH1* mt and *IDH1* wt mean probe 5hmC *β*-values of 4.6%, and 3.7%, respectively. Conflicting reports exist regarding the influence of *IDH1* mutation on overall 5hmC abundance in gliomas. Reduced immunopositivity for 5hmC by immunohistochemistry (IHC) has been observed in small cohorts of *IDH1* mt tumors [39, 75]. Others studies noted no correlation between *IDH1* status and overall 5hmC levels using IHC or liquid chromatography–mass spectrometry [26, 32, 33, 48, 53]. One caveat to our approach was that the MethylationEPIC BeadChip interrogated only a subset of cytosine loci and may not have been representative of whole genome 5hmC abundance. An advantage over previously reported methods, however, was the ability to compare 5hmC in *IDH1* mt and *IDH1* wt tumors quantitatively in a region-specific manner. For probes in regions with the greatest 5hmC abundance (top 1% probes) or differential hydroxymethylation (DHMR probes), *IDH1* mt tumors demonstrated *higher* levels of 5hmC (Fig. 1).

While this finding is counterintuitive based on a model of reduced demethylation secondary to *IDH1* mutant-mediated TET inhibition, other mechanisms exist that influence cellular homeostasis between 5mC and 5hmC. TET enzyme expression and subcellular localization have been shown to influence 5hmC levels in gliomas [48, 53, 67]. *TET1*-*3* expression did not correlate with 5hmC levels in our cohort, and subcellular localization of TET family enzymes was not examined. Correlating additional epigenetic enzyme expression (*IDH1/2*, *DNMT1*, *DNMT3A/B*) and mean tumor 5hmC *β*-values demonstrated a significant negative correlation between *DNMT3B* expression and 5hmC when considering loci of high 5hmC abundance (top 1% probes, *r*_s_ = − 0.603, *p* value = 0.0049) (suppl. Table 13, Online Resource 24). While *DNMT3B* is classically viewed as a de novo DNA methyltransferase, it has been identified as possessing 5hmC *dehydroxymethylase* activity as well, providing another pathway for conversion of 5hmC to C [9]. The fact that multiple factors impacting 5hmC levels have been identified may partly explain the existence of a third mixed cohort of *IDH1* mt and *IDH1* wt tumors seen when samples were clustered based on 5hmC in our study.

5hmC accumulation at enhancer and super-enhancer regions has been reported [57, 73]. Johnson et al. recently demonstrated enrichment of 5hmC targeting enhancers and super enhancers in *IDH1* wt GBM [28]. Our data confirmed this finding and further extended it to include *IDH1* mt tumors. Despite this common feature, we observed only partial overlap of enhancer and super enhancer regions marked by 5hmC in our *IDH1* cohorts. Cancer cells may acquire super enhancers associated with oncogenes such as *MYC* that are not present in normal cells [23]. In the GBM cell line u87, super enhancers targeting *CCND1*, *CDK6*, *EGFR*, *JUN*, *MET*, *MYC*, *NOTCH2* and *RUNX1* have been identified [23]. In our study, super enhancer targets marked by 5hmC in common between *IDH1* mt and *IDH1* wt tumors and previously implicated in gliomagenesis included *EGFR*, *MYC*, *CDK6*, *NOTCH2*, *RUNX1*, *PDGFRB*, *PXN*, *ID3*, *IGF1R*, *NEDD9*, and *MSI2* [13, 27, 34, 42, 50, 58, 64]. Additional *IDH1* wt super-enhancers marked by 5hmC in our dataset and implicated in GBM pathogenesis included *WDR1*, *TGFBI* and *PVT1* [11, 36, 43, 74]. *IDH1* mt-specific super-enhancer targets marked by 5hmC in our cohort and previously implicated in GBM pathogenesis included *PDGFC*, *PRRX1*, *LIF*, A*XL*, and *CD44* [14, 18, 31, 50, 55, 63, 71]. Taken together, these data demonstrated enhancer/super-enhancer targeting by 5hmC as a prominent feature in HGG, with differential targeting observed between *IDH1* cohorts.

G-CIMP status is a characteristic feature of *IDH1* mt gliomas. To date, the relative contributions of 5mC and 5hmC to overall methylation of G-CIMP genes have not been examined. Our data demonstrated that unsupervised hierarchical clustering based specifically on 5mC *β*-values from OxBS-treated samples distinctly separated *IDH1* mt from *IDH1* wt tumors. Despite this, a subset of G-CIMP genes were marked by 5hmC in *IDH1* mt tumors, adding to the overall methylation levels measured when using bisulfite conversion-based methods alone. As *IDH1* mutation-generated 2-HG inhibits TET-mediated active demethylation, one may expect a strong anti-correlation between probe level 5mC and 5hmC levels, in particular for probes targeting G-CIMP defining genes. Interestingly, we observed the degree of negative correlation between 5mC and 5hmC for G-CIMP genes to be significantly *lower* than that seen across all probes. One possible explanation for accumulation of both 5mC and 5hmC at G-CIMP gene loci may relate to differential affinities demonstrated by TET1 and TET2 for 5mC versus 5hmC as substrates [25]. Impaired TET activity secondary to *IDH1* mutation may have a greater impact on the downstream conversion of 5hmC to 5fC, compared with 5mC to 5hmC. Reduced oxidation of any 5hmC generated may lead to its gradual accumulation over time alongside 5mC at G-CIMP loci.

The mechanisms by which 5mC and 5hmC regulate gene expression are not fully understood; however, some common themes have emerged. DNA methylation (5mC) has traditionally been viewed as an epigenetic silencing event [29]. With improvements in the ability to map 5mC genome wide, its influence on gene expression is now known to exhibit contextual differences depending on the region targeted. Promoter-region CpG island methylation generally correlates negatively with gene expression [29]. The influence of gene body methylation on gene expression remains controversial, with both facilitation and repression of transcription reported in the literature [30, 40, 69, 77]. The use of bisulfite-based methods to quantify gene body methylation when examining its role with respect to gene expression may contribute to the varied results reported. In such cases, the individual contributions from 5mC and 5hmC would not be distinguishable. With respect to 5hmC, gene body hydroxymethylation has been associated with increased transcription [29, 51, 73]. Based on 5mC, 5hmC and gene expression data from our tumor cohorts, we observed 5mC targeting regions flanking the transcription start site (TSS200/1st exon probes) to be associated with reduced gene expression. Gene body 5mC was neither strongly nor consistently associated with gene expression. With respect to hydroxymethylation, we identified gene expression to be most strongly associated with 5hmC targeting gene body regions, in keeping with prior reports [12, 17, 38]. At the gene-specific level, using two separate approaches (examining regions of high 5hmC abundance as well as DHMRs), we observed a highly significant striking pattern of positive correlation between 5hmC and gene expression for genes that are highly expressed in the *IDH1* mt cohort (Group 1 probes). In addition, the majority of Group 1 probes targeted gene body regions, with the *IDH1* mt tumors almost invariably possessing higher mean 5hmC *β*-values compared to *IDH1* wt tumors. This raises the possibility of 5hmC facilitating increased transcription for this subset of genes in *IDH1* mt tumors. With gene body 5hmC associated with increased expression, and the trend towards accumulation of 5hmC observed in *IDH1* mt tumors, an intriguing possibility exists that epigenetic dysregulation of gene expression in *IDH1* mt gliomas arises, in part, due to impaired oxidation of 5hmC to 5fC across gene bodies, facilitating expression of target genes.

One putative target gene identified in our cohort was *LGR5*—a downstream target of *WNT* pathway activation that has been implicated in promoting a cancer stem cell (CSC) phenotype in GBM [22, 44, 49, 72]. Elevated *LGR5* expression has been correlated with increasing tumor grade and reduced OS [49, 72]. Knockdown of *LGR5* cells reduced proliferation and tumor sphere formation in vitro, and impaired tumor formation in vivo [72]. Mao et al. demonstrated preferential expression of *LGR5* in proneural GBMs, consistent with our finding of increased expression in the *IDH1* mt cohort [44]. Epigenetic regulation of *LGR5* in gliomas has not yet been described. In our study, we observed increased expression of *LGR5* among *IDH1* mt tumors. A highly significant correlation between 5hmC *β*-value and gene expression was identified, raising the possibility that *LGR5* expression in glioma may be regulated epigenetically by 5hmC.

To conclude, we describe, for the first time, a quantitative locus-specific analysis of 5hmC in *IDH1* mt HGGs compared with *IDH1* wt tumors. Enhancer and super-enhancer targeting by 5hmC in *IDH1* mt tumors was identified, targeting genes implicated in GBM pathogenesis. A paradoxical increase in 5hmC for regions marked by high 5hmC abundance and DHMRs was observed in *IDH1* mt tumors. The correlation between 5hmC and 5mC was greater than anticipated for G-CIMP gene probes based on that expected by a model of 5mC accumulation secondary to reduced TET-mediated oxidation to 5hmC alone. A significant correlation between gene body 5hmC and gene expression was identified, including a striking association between increased 5hmC and genes upregulated in *IDH1* mt HGGs.

## Electronic supplementary material

Below is the link to the electronic supplementary material.

Supplementary material 1 (XLSX 38 kb)

Supplementary material 2 (EPS 3480 kb) Suppl. Figure 1 (Online Resource 2) 5hmC *β*-values compared with OxyBS package a) Correlation between probe level 5hmC *β*-values between our analysis (BS-OxBS) versus OxyBS R package for *IDH1* mutant tumors. b) Correlation between probe level 5hmC *β*-values between our analysis (BS-OxBS) versus OxyBS R package for *IDH1* wild-type tumors. c) Venn diagram depicting overlap between top 1% probes identified by our analysis (BS-OxBS) versus OxyBS R package for *IDH1* mutant tumors. d) Venn diagram depicting overlap between top 1% probes identified by our analysis (BS-OxBS) versus OxyBS R package for *IDH1* wild-type tumors

Supplementary material 3 (EPS 2177 kb) Suppl. Figure 2 (Online Resource 3) Representative Chromatograms for *IDH1* and *IDH2* Sequencing a) Examples of representative chromatograms depicting *IDH1* wild-type (“IDH1 normal”), as well as two *IDH1* mutant variants (“IDH1 R132H mutant” and “IDH1 R132C mutant”). b) A representative chromatogram depicting *IDH2* wild-type (“IDH2 normal”) sequencing

Supplementary material 4 (XLSX 83 kb)

Supplementary material 5 (EPS 2574 kb) Suppl. Figure 3 (Online Resource 5) Genomic abundance of 5hmC and 5mC, and distribution related to CpG island features, in *IDH1* mutant and wild-type high-grade gliomas a) Cumulative density plot across all probes for 5hmC *β*-values for *IDH1* mutant (*IDH1* mt) versus *IDH1* wild-type (*IDH1* wt) tumors. b) cumulative density plot across all probes for 5mC *β*-values for *IDH1* mt versus *IDH1* wt tumors. c) and d) Cumulative density plots for 5mC *β*-values stratified by CpG island feature, demonstrating overall reduced CpG island 5mC and greater 5mC content targeting CpG island shores and shelves for *IDH1* mt and *IDH1* wt tumors, respectively. e) and f) Cumulative density plots for 5hmC *β*-values stratified by CpG island feature, demonstrating overall reduced CpG island 5hmC and slightly greater 5hmC content targeting CpG island shores and shelves for *IDH1* mt and *IDH1* wt tumors, respectively

Supplementary material 6 (XLSX 1490 kb)

Supplementary material 7 (EPS 2574 kb) Suppl. Figure 4 (Online Resource 7) Distribution of top 1% 5hmC and 5mC probes, and DHMR probes, stratified by CpG island feature and genomic regions. a) Distribution of top 1% 5hmC probes stratified by CpG island features, expressed as percentage of total number of top 1% 5hmC probes. b) Distribution of top 1% 5mC probes stratified by CpG island features. c) Distribution of top 1% 5hmC probes stratified by genomic regions as annotated by Goldmine, expressed as percentage of total number of top 1% 5hmC probes. d) Distribution of top 1% 5mC probes stratified by genomic regions. e) Distribution of DHMR probes in relation to CpG island features. f) Distribution of DHMR probes in relation to genomic regions

Supplementary material 8 (XLSX 873 kb)

Supplementary material 9 (EPS 2878 kb) Suppl. Figure 5 (Online Resource 9) Pathway analysis for top 1% and DHMR probe targets a) Venn diagram depicting the degree of overlap versus unique probes among the top 1% 5hmC probes in *IDH1* mutant and *IDH1* wild-type tumors. b) Pathway analysis for top 1% 5hmC probe targets. Unique and common pathways identified among the top 10 pathways for each of *IDH1* mutant and *IDH1* wild-type tumors are listed, along with the number of matching genes observed for each pathway. c) Pathway analysis for differentially hydroxymethylated region (DHMR) 5hmC probe targets

Supplementary material 10 (XLSX 14 kb)

Supplementary material 11 (EPS 2179 kb) Suppl. Figure 6 (Online Resource 11) Enhancer targeting based on primary human GBM tumor propagating cell line enhancer annotations. Additional data demonstrating ORs for enhancer targeting of DHMR probes as well as top 1% probes (all tumors, *IDH1* mt, *IDH1* wt) based on enhancer annotations from H3K27Ac ChIP-seq data on the a) MGG4 and b) MGG8 tumor propagating lines

Supplementary material 12 (XLSX 13 kb)

Supplementary material 13 (EPS 2286 kb) Suppl. Figure 7 (Online Resource 13) 5hmC-based clustering identifies co-segregation of a subset of *IDH1* mutant and wild-type tumors. Consensus clustering based on 5hmC *β*-values for overlapping DHMR and top 1% probes (*n* = 321) demonstrates 3 robust clusters. Cluster 1 consists solely of *IDH1* mt tumors, while cluster 3 consists of *IDH1* wt tumors. Cluster 2 is mixed with both *IDH1* mt and wt tumors. WHO grade III/IV represents tumors classified pathologically as “high-grade glioma”

Supplementary material 14 (EPS 2352 kb) Suppl. Figure 8 (Online Resource 14) 5hmC Consensus clustering CDF and silhouette plots. a) Consensus CDF and b) silhouette plots, demonstrating stability of 3 cluster groups resulting from consensus clustering for all samples based on 5hmC *β*-values. For comparison, results for 2 cluster membership are depicted in c) and d) demonstrating less robust cluster membership

Supplementary material 15 (XLSX 84 kb)

Supplementary material 16 (XLSX 125 kb)

Supplementary material 17 (XLSX 35 kb)

Supplementary material 18 (EPS 3374 kb) Suppl. Figure 9 (Online Resource 18) 5hmC versus gene expression by gene region. a) Notched box plot depicting *IDH1* mt top 1% probe correlation with annotated target gene expression, stratified by probe gene region. b) Notched box plot depicting *IDH1* wt top 1% probe correlation with annotated target gene expression, stratified by probe gene region. c) Distribution of top 1% probes demonstrating a significant Spearman correlation with genes differentially expressed between *IDH1* mt and *IDH1* wt tumors, divided by gene region

Supplementary material 19 (XLSX 15 kb)

Supplementary material 20 (XLSX 151 kb)

Supplementary material 21 (EPS 1306 kb) Suppl. Figure 10 (Online Resource 21) *LGR5* gene expression correlates with hydroxymethylation. a) Significant strong positive Spearman correlation between 5hmC *β*-values and gene expression for candidate gene *LGR5* (probe ID cg23900866). b) Non-significant Spearman correlation between 5mC *β*-values and gene expression for the same probe

Supplementary material 22 (XLSX 1043 kb)

Supplementary material 23 (EPS 2028 kb) Suppl. Figure 11 (Online Resource 23) hMeDIP-PCR validation of select gene candidates. hMeDIP-PCR was performed on an expanded cohort of *IDH1* mt (*n* = 25) and *IDH1* wt (*n* = 16) tumors, for gene candidates demonstrating significant positive Spearman correlation with gene expression, for genes differentially expressed between *IDH1* mt and *IDH1* wt tumors. For each candidate, 5hmC was increased among the *IDH1* mt tumor cohort based on Illumina MethylationEPIC array data. Primers flanking the respective Illumina probe loci were used for PCR amplification. Enrichment following hMeDIP is demonstrated among the *IDH1* mt cohort for a) *LGR5,* b) *WDR11*-*AS1,* c) *CRTAC1*, d) *GRID1,* and e) *FAM155A*

Supplementary material 24 (XLSX 14 kb)
